# The female spouse: A process of separation when a husband 'comes out' as gay

**DOI:** 10.1371/journal.pone.0203472

**Published:** 2018-08-30

**Authors:** Siobhán C. Daly, Pádraig MacNeela, Kiran M. Sarma

**Affiliations:** School of Psychology, National University of Ireland Galway, Galway, Ireland; The University of Warwick, UNITED KINGDOM

## Abstract

This study investigated the stories of heterosexual women who experienced a husband coming out as gay and a consequential marital separation. Interpretative phenomenological analysis (IPA) was used. Loss, anger, spousal empathy and concerns regarding societal prejudice were reported. Additional stress was experienced when others minimised the experience due to the gay sexual orientation of their husband. Experiencing positive communication with their husband during and after the disclosure aided the resolution of the emotional injury experienced by them. They all eventually ‘let go’ of their husband. This involved a process of reconceptualising the self as separated. Findings indicate the importance of supporting women to re-focus on their needs during and following marital dissolution. The importance of non-judgemental support for marital loss, rather than a focus on the gay sexual orientation of the spouse, was highlighted.

## Introduction

When a husband ‘comes out’ as gay (accepting and revealing oneself as gay), it impacts the family unit. Yet little is known about the experiences of the heterosexual spouse. Marriages where one partner comes out face the potential stigma of a ‘failed marriage’ in addition to the possibility of homophobia [1]. Stress and anxiety regarding the implications of a gay identity being at odds with their religious faith or community of the couple can occur [2, 3]. The altered sexual orientation identity of a spouse may include changes in behaviour, group affiliation, personal values and norms [4, 5]. Moreover, a fear of rejection by family, friends, or a religious community can result in suppression and isolation, especially in more conservative or homophobic environments [6, 7].

It seems reasonable to assume that the experience of a spouse having same-sex desires resonates with families within which extra-marital affairs occur (e.g. husbands with other women). For the wife, these would include distress at the rupture in the emotional bond with her husband [8] and feelings of betrayal [9]. More broadly, concern for children, emotional turmoil and loss, financial uncertainty, changing family and social ties and loss of, and desire for, companionship, may arise as they do in any marital crises [10, 11]. Marital dissolution rates are high across Western countries [12] and support is important in negating the negative health consequences of separation or divorce, if that is the outcome [13].

Some couples may wish to maintain their marriage and seek to accommodate the spouse’s same-sex romantic or sexual attractions [14]. A lifelong marital commitment perspective assumes the dedication of each spouse to the other and acknowledges that marital conflict can often occur [15]. Sexual needs may be met outside of the marriage with an agreement to engage in separate sexual relationships (non-monogamous), adding another layer of complexity to the situation [16]. While a consensual non-monogamous marriage can be a preferable path for some couples, others may engage in hidden infidelity, and have secret, anonymous sex with multiple partners [17]. Such secret infidelities may strain the emotional well-being and physical health of the spouses [18].

The loss of a happy marriage in later life may be experienced akin to a spousal bereavement, with associated rates of depressive symptoms, especially for those who value their marital identity [19, 20]. However, some studies suggest that required support may be perceived as unavailable, due to family or religious homophobic beliefs [21, 22]. Yet such support is important for couples in acute distress to aid their processing of the disclosure and reduce feelings of social isolation and depression [23].

To date, many of the assumptions and assertions of the heterosexual spouse are based on disparate sources of information from the literature on the experiences of a spouse ‘coming out’. The findings in this niche area are largely unsystematic and tend to comprise personal or anecdotal case descriptions, or when empirical are primarily descriptive. A small group of published studies, for example those carried out by Amity Buxton (e.g. [24, 25]) have provided insight into common issues reported by the heterosexual spouse, such as marital challenges, isolation, concern for their children and self, and crises of identity and belief systems. Where couples decide to continue to reside together, it can be difficult to know what should be disclosed by the couple, and at what stage this disclosure should occur [26]. Further research is required to explore the experiences of the heterosexual spouse, and how a marital dissolution following a same-sex affair differs from that of a heterosexual affair.

This study sought to expand on the existing literature, and on research carried out by the authors, which explored the experiences of husbands who came out as gay in the context of a heterosexual marriage (publication forthcoming), and children who had a parent come out as lesbian, gay or bisexual. The latter study indicated that the primary focus of the participants was in adjusting to their parental separation [27]. In this study, the lived experiences of mothers and wives, whose voices may appear more silent in the context of a disclosure of a gay sexual orientation of their spouse, were explored. An interpretative phenomenological analysis (IPA) approach was adopted in seeking to understand the unique experiences of having a husband disclose as gay following a marriage that produced children. The IPA methodology focused on how each person made sense of this specific phenomenon [28].

## Methods

The researchers were interested in how wives made sense of their personal experience of their husband coming out to them as gay. IPA was the chosen methodology due to its ability to offer insights into how people make sense of a lived experience [29], especially in the context of a significant life change (i.e. the disclosure of a gay sexual orientation that changed the marital relationship) and a focus on the self (as wife, mother, individual). IPA focused attention on how the women recalled, retrospectively, the significant transition and disruption that occurred in their marriage (a phenomenological process; [30]). It allowed the researchers to try to interpret the participants trying to make sense of their experiences (a double hermeneutic process; [28]). IPA also accommodated the individuality of each person through an in-depth analysis of each singular case (an idiographical process; [31]).

### Participants

All women had (a) experienced a husband come out as gay, and (b) had a child or children with their husband. Data collection ended after completing the ninth interview due to the richness of the individual cases. IPA sampling tends to be small (usually fewer than 10 participants) and seeks homogenous groups of participants. The focus is on the individual [32]. As IPA is idiographic in nature, it focuses on the unique, personal experiences comprising the phenomenon under investigation before analysing convergences and divergences between cases [30]. The study focuses on the accounts of nine participants whose contextual information is presented in Table 1. Names have been changed to protect their identity. They ranged in age from 49 to 62 years (mean age = 54.5 years) and all identified as heterosexual. Six were Irish, one was Scottish and two were Canadian. They were aged between 18 and 25 years when they got married and the mean marital length was 26 years. Their children at time of interview ranged in age from 13 to 41 years (mean age = 25.5 years). The mean length of time from disclosure to marital separation was seven years. Four were divorced, three had commenced the legal process of divorcing and two were ‘separated’. All the participants had to make sense of what the disclosure meant for their marriage and themselves. This was the key focus of the study.

**Table 1:** Contextual information of participants.

Pseudonym	Age	Marriage length	Time from disclosure to separation
Mary	54	30–35 years	5 years
Helen	50	20–25 years	2 years
Sarah	49	15–20 years	7 years
Rose	62	20–25 years	5 months
Lorraine	51	20–25 years	6 years*
Patty	60	20–24 years	29 years
Grace	50	20–24 years	14 years
Lucinda	58	30–35 years	7 years
Christine	57	30–35 years	5 months

Note.

* Lorraine separated from her husband six years ago. They continue to cohabitate.

### Procedure

Full institutional ethical approval was obtained from the National University of Ireland Galway (NUIG) Research Ethics Committee before the study commenced. An email detailing the recruitment details of the study were sent to members of the Irish Association for Counselling and Psychotherapy, and the Straight Spouse Network (SSN). Two participants heard about the study from their ex-husband, three via word-of-mouth, one was informed of the study by a therapist, and three (non-Irish) responded to information disseminated via the SSN. The participants volunteered, making contact with the first author directly by telephone or email. The entire research was discussed with each participant who received an information sheet about the study. They completed a consent form prior to being interviewed, and consented to the use of their anonymised interviews for analysis and publication. They were interviewed in their homes when it suited them.

The first author completed the interviews and the analysis coding. Each interview was audio recorded. Six were face-to-face in Ireland and the remainder (n = 3) abroad, via Skype. Interviews lasted up to two hours (modal length = 80 minutes) and were open-ended. In line with the IPA approach, questions focused on each unique experience of having a husband ‘come out’ and the consequential changes that occurred. Topics focused on during the interview included: sexual experience(s); experience of marriage before the disclosure; any signs/awareness of changes in the marriage and/or husband; the disclosure; consequential impact on the family unit and self; telling others; the most difficult thing(s); sources of support; subsequent relationships and current relationship with husband or ex-husband.

Critical self-reflection (reflexivity) is required in IPA, as researcher presuppositions, experiences and beliefs can both inhibit and augment the interpretations of the experiences of the participants. The first author has a father who identifies as gay. She drew on her experience as a psychologist in interviewing people about potentially sensitive topics, and was cognisant of the potential impact of her own assumptions on the research process. Reflective memos made during the study were carefully considered as the interpretative process proceeded. They also served as a method of debriefing. A further strategy used was to discuss, confidentially, the (anonymised) arising themes and individual differences within the accounts at supervisory research meetings. The second author is a cisgendered male who was socialised into traditional Irish culture dominated by the Catholic Church and the lifelong pattern of marriage described by the participants in this study. He has also seen how this model has been questioned in recent decades. He has extensive experience of working on qualitative projects in which participants reflect on traumatic life changing circumstances that cause them to question their basic assumptions. He has developed a particular interest in sexual health research in recent years and promotes culture change based on open discussion of preferences within a culture of mutual respect. The third author is a married heterosexual male of mixed Irish-Indian heritage. He has worked closely with the LGBT community on issues relating to homophobic bullying, mental health, peer support and victimisation.

Jonathan Smith’s IPA evaluation guide [29] informed the iterative and inductive analysis process. Each audio recording was transcribed and read several times to gain a more holistic understanding of the depth of the account. The primary author transcribed each interview and analysed the nuances of each account (a case-by-case analysis). This was followed by a phase of comparing and contrasting the accounts. Initially, meaning units or codes were identified by reviewing the transcripts, line-by-line open coding, noting thoughts next to the corresponding text, and writing a description of the experience (focusing on emotions, phrases, places, metaphors, actions). Emerging themes were established for each case. Descriptions were translated into psychologically relevant meanings by moving back and forth from data to meanings, while also integrating the researcher memos and descriptive interpretations. Themes and subthemes began to emerge.

The second and third author reviewed the transcripts and the arising thematic interpretations. The iterative process was discussed collaboratively at supervisory meetings, and reflections on the different nuances arising from (each and across) the accounts occurred. The codes were examined for relevancy with regard to the research question, and discarded if deemed irrelevant. Conflicting perspectives were utilised by exploring the contexts of differing experiences, and constructing a portrayal of how the phenomenon was also experienced, individually [33]. Broader themes were identified, drawing upon psychological concepts and examining the nuances of each superordinate theme. The main themes were solidified into a final structure that seemed to best summarise the data. Participant quotations were used to illustrate the essence of the themes being recounted. Care was taken to include a sufficient range of sampling when evidencing each theme, in accordance with IPA guidelines [29,34]. Finally, an account summarising the interlinking activity of the researchers and the participants’ interpretations was produced. The aim was to provide an understanding of how the participants experienced key factors that emerged–making sense of a husband disclosing as gay’.

## Results

A summary of the main results is given in Table 2. Three main themes emerged: ‘Committing to lifelong marriage’, ‘Marital floundering and limbo’ and ‘Having to move on’.

**Table 2:** Superordinate and subordinate themes.

Thematic Number	Thematic Name
Theme 1	Committing to lifelong marriage
Theme 2	Marital floundering and limbo: being partially married, partially separated
Subtheme 2.1	Loss, anger and empathy
Subtheme 2.2	The fear of stigma
Subtheme 2.3	Adjusting the marital script
Theme 3	Having to move on (living apart)
Subtheme 3.1	The marital end: crossing the Rubicon
Subtheme 3.2	Self-integration: ‘salvage what’s good and move on separately’

### Theme 1: Committing to lifelong marriage

This short theme is an overview of the participants’ hopes for, and experience of, their marriage prior to their marital difficulties. Marriage fitted the idealised picture they had imagined and believed during their youth. Phrases such as falling “head over heels in love”, or falling “‘hook, line and sinker” exemplified the deep love they recalled towards their husband. Nearly all (n = 8) surrendered their careers outside the home to take charge of their role as home maker and wife. Rose described how as a young adult she believed she had found her match and remembered proudly herself being competent in her role of mother and wife: “I followed my heart’s desire and when I was 18 we married. I loved the life of a housewife and mother. I truly thought that marriage didn’t come any better than what we had. Everyone admired our marriage and I was in love and felt love”.

The religious background of the participants (eight identified as Catholic, one as non-defined) reinforced the assumption that “you married for life”. Patty described how she was happy to conform to social and religious traditions to please her parents, to legitimise sex and have children. In keeping with her faith, which viewed premarital sex as sinful, she (and participants n = 7) remained chaste until her wedding night. She dedicated herself to her marriage; it was both a splendid reality and serious business: “I was a traditional Catholic. What lay ahead—marriage, sex, kids- seemed thrilling. Only the wanton ones were having sex before marriage back then. That was the prevailing culture. I married and vowed to stick with my husband”.

All the participants initially presumed their marriage would continue to develop over their life. Most either gave up work (n = 6) or reduced their career hours (n = 3) when they married and had children. Helen recalled a lifetime of experiences with her husband, namely establishing a home, having children, and supporting each other in times of spousal ill-health or following the death of significant loved ones. She surrendered her career outside the home to take charge of her role as home maker and wife. Her use of the term ‘golden years’ seems to reinforce the hope that the idealised picture would continue and she could make the most out her marriage later in life, travelling as a celebratory reward: “I gave up work and managed our home. We were married for over 20 years and went through everything together. I thought I’d spend my retirement with him, my golden years, travelling the world, visiting our children”.

### Theme 2: Marital floundering and limbo (being partially married, partially separated)

Following a lengthy period of relative marital stability, unexplained tension and a sense of disconnection with their husband was described. Helen noted changes in her husband’s mood (“he seemed more switched off and agitated.”). A distancing within their sexual relationship was a worrying indicator that there was something wrong: “When we eventually did have sex I remember thinking he was more athletic, that there was something different. And one night when he didn’t come home I realised that in the back of my mind I asked ‘did he pick up a rent boy?’ I had it, but I didn’t want to think about it.” Her husband’s new found athletic potency contrasted with the inactive and weakened connection between them. She started debating her husband’s sexuality internally, but ‘did not want to believe it’ and relegated the thought that there was something wrong to the back of her mind.

#### 2.1 Loss, anger and empathy

The narrative threads of the disclosure sequence were ones of increasing intensity of feeling, and progression into the verbal confrontation after the disclosure. Despite their suspicions, the disclosure was experienced by them as abrupt, dramatic, penetrating and dislocating. Emotional pain was felt bodily: “It felt like I’d been kicked in the stomach. I felt ice-cold” (Christina); “It was like a slap to the face” (Grace). Words such as “zombie”, “autopilot” and “blurry” conveyed a sense of shock and a loss of connection. Participants described something akin to an existential crisis: the realisation that the ‘secure home and marriage’ was paradoxically unstable, causing significant distress. Nearly all did not want to be separated, nor for their husbands to want intimacy with others. Many years later, the loss of her marriage still triggers painful tears for Mary: “When he told me the tears came and they just did not stop. I was absolutely devastated. Heart-broken. I still love him [upset]. We were married for over 30 years. This wasn’t the plan. I never thought I’d be on my own. That was the hardest part”.

Mary, and all the participants, expressed anger towards various significant others including family, friends, God, and society at the marital breakdown. However, she felt empathy towards him. Seeing and hearing her husband ‘struggle’ to accept his sexuality quelled feelings of anger that arose towards him, and also forced her to accept his gay identity. Even after the separation, echoes of empathy continue–although she is angry at her loss, her frustration towards her husband is tempered by a continuing concern for his well-being: “He told me he had gone up to the attic with a rope. He was going to hang himself. I never showed him anger because I didn’t think he deserved it. But God I have been angry, because he put me in this situation. I still care for him and want him to be happy.” This effect was evident across the narratives, including anger and disappointment directed towards the self: “How could I have been so stupid; He can’t help it.” (Helen)

Patty’s process of finding meaning in the origins of her husband’s gay identity resulted in an understanding that the disclosure was not, fully, her husband’s fault. She believed his reasons for not disclosing his same-sex desires when they wed, which he shared with her. This appeared to enable a continued dialogue between them. Rather than focusing on her choices and constructing an anticipated future life as separated, Patty initially focused on her husband:

He told me he had talked to the GP [doctor] about having thoughts about men before we got married. He said ‘don’t worry that’s quite common. Once you get married and you start having sex with her all of that will just fade away’. He thought, ‘That’s what I want to hear’. To be gay for him it was a hellish sin. It wasn’t all his fault; society is a lot to blame.

In contrast, Grace’s husband did not discuss the origins of his gay sexual orientation with her, and he believed that his extra-marital affairs were unrelated to their marriage. She expressed anger towards him, which increased when he ‘outed’ his gay identity to others (and their troubled marriage), without her knowledge or consent, and which she considered a betrayal of their union. The disclosure threatened her own assumed safe world. Grace did not want to be a divorcee. She tried to make him accountable for his actions (“being with guys is having an affair”), but he had stopped listening. The resultant hostile silence between them was never repaired.

He never talked to me about why, or thought of my feelings. I was ‘outed’ by him. He told everybody in work. I will admit I have been very nasty and angry. I felt so betrayed. I tried to explain to him, ‘it’s not that you are gay; it was your behaviour’. But he wouldn’t hear me. It is difficult to be separated and not want to be.

Grace’s feeling of anger was further compounded when their marriage counsellor focused on her reasons for staying in her marriage; ‘He kept asking me ‘why didn’t you leave years ago?’. The participants, like Grace, felt frustrated when others advised them to separate: ‘He didn’t get it or understand how hard it was to have young children and not want them to be torn between two parents’. Her perception of her counsellor as judgemental resulted in her disengagement from therapy; no alternative ‘script’ was offered. Like Grace, the majority of the participants (n = 7) sought professional support with their husband at the time of the disclosure, but they did not find it helpful. They were often urged to ‘move on’, which was not in keeping with their desires, or were offered sexual advice that focused on their husband’s sexuality. This resulted in further frustration.

Nearly all the participants (n = 8) hoped that their marriage could be maintained. Having very young children, unwell adult children, financial concerns, and spousal ill health significantly lengthened the separation process. Furthermore, it was difficult to consider separating when the couple communicated to one another that they did not want this to occur (n = 7).

#### 2.2 The fear of stigma

Love and empathy coupled with grief and the shattered ‘marital sanctuary’ seemed to instigate the participants’ constant worry about their family and their future self. Anxiety resulted from the negative social stigma they had traditionally associated with divorce and homosexuality. Helen worried “will it turn people? Will everybody be looking at me, thinking it was a marriage of convenience?” She feared the disclosure would result in a negative evaluation of herself and her family, subsequently resulting in social exclusion: “being the talk of the town”. Sarah worried about her children being impacted by the societal prejudice–she was reared in an era where the cultural taboo of divorce and homosexuality were societal sins: “I was so worried about the kids. That they would be teased. Fellas can be cruel and say things like ‘feck off your father’s a faggot’. There are a lot of people in heterosexual relationships who stay together for the family life. In our era you got married for life.” The stigmatising aspect of the marital separation is evoked, with the added element of societal and sexual prejudice. Sarah’s reluctance to separate was further exacerbated by protective, parental feelings that arose when she thought of others possibly negatively evaluating, or teasing, her children.

All of the participants shared their need to be understood by significant others while experiencing the marital dissolution. However, validation of their feelings initially from others was often limited, or unavailable. Patty highlighted the stress she experienced in withholding her marital difficulties from friends: “I couldn’t be with my friends and not be fully open. It felt like daggers. You’d be there smiling and pretending. We used to share everything. So it was easier to not meet them.” The deception and pain appeared to reinforce each other, making it harder and harder to face what was really going on. Withholding information for Patty meant she had to think of what she was trying to hide (marital problems) and deliver a convincing performance of the opposite (marital normality). The pain of pretending felt something akin to being stabbed. That was unbearable and so, for a few years, she avoided her peers.

#### 2.3 Adjusting the marital script

With their desire to remain married, and the stigma they internalised about the prospect of change, the participants described the process of becoming separated as slow and incremental. Nearly all (n = 8) did not sleep in the same bed again once they or their husband had disclosed about being intimate with another man. While most participants described themselves as dedicated to their husband, three participants, and most of the husbands, engaged in extra marital affairs, which provided some degree of escape and enabled the marriage to continue.

Patty tried to tolerate a more consensual non-monogamous marriage so as to avoid the family being a ‘broken home’. However, sexual relations outside the marriage caused further confusion and dissonance for her given her religious beliefs. In this extract there is a strong sense of the internal ‘whirling’ and ruminative distress that she experienced:

I was in a sea of confusion. One part of me was saying ‘God closes a door and opens a window’, and this is my window. On the other hand my upbringing was telling me ‘you can’t do that; what are you doing?!’ We had done our deal—we would stay together and be a priority for each other. But he reneged on that deal. But he still needed me.

Patty tried to maintain a pre-disclosure version of their marriage which was transactional (“our deal”) and caring (“he needed me”). The extramarital affair was going against her religious beliefs and the marital identity to which she had originally committed, and contradictory ambivalence was aired in simultaneously experienced opposing thoughts (rebellion and obedience, vice and virtue).

The consequences of trying to make things work gave rise to further feelings of instability and some of the participants (n = 3) fantasised that an event outside their control would ‘respectfully’ force the change from married to separated (via an accidental death), without having to go through the process. For Lucinda there was a metaphorical sense of the walls of her marital home closing in, as she struggled to maintain her fragmented identity together. She eventually acted on her escapist thoughts–she left with ‘nothing’, which may have reflected the marital void: “Eventually I thought ‘I can’t do this anymore. I can’t live this lie'. I needed to get out—it was all closing in. I walked out, with nothing but my car and my handbag. I had to.” However, for Lorraine the marital home was a critical factor in maintaining her marriage. She was the only participant who remains united with her husband. She continues to support and depend on him and although she is exploring career and dating interests outside the home, she is limited in how far she can expand on these. Her use of the word ‘We’ instead of ‘I’ reaffirms their intertwined (almost telepathic) connection: “I’ll just look at my husband and we both know what we’re thinking. We’re good friends. We have our home. Someday we should get divorced, but I’m not financially secure and I have health issues”.

In contrast to all the other participants, Christina bypassed the process of marital limbo and the consequential angst or dissonance involved in being partially married and partially separated. She decided to separate soon after the time of disclosure, despite her loss and her husband’s reluctance and sorrow. Her account highlights refusal (“not going to live with”), openness (“come out into the light”) and separation (“we have to break up now”). Although Christina empathised with her husband, she did not want to identify with a marriage that involved ‘others’ or any pretence:

I knew at that moment exactly what I was going to do. That I was going to separate from him, although I loved him dearly and still do [upset]. He didn’t want to tell the kids but I said, ‘we have to be open and tell them, because I’m not going to live in a closet with you. We are going to come out into the light and we are going to own this’. He felt terrible, but I knew I didn’t have a choice and that my life was changing irrevocably. And there was no turning back.

While Christine moved relatively quickly to end the marriage, most participants did not. They expressed despair and anger which were vented in different directions, including anger towards self. Anger was often coupled with empathy towards their husband in ‘coming out’, if they were communicating with one another. They questioned the self and others, seeking to make sense of their broken marriage and to relieve the tensions from their being partially married/partially separated. Support was not found to be helpful at this time.

### Theme 3: Having to move on (living apart)

Eight of the participants showed clear signs of being loathe to end the marriage. This theme focuses on the actual transition and irrevocable step (‘crossing the Rubicon’) that resulted in every participant, except one, identifying as fully separated and living apart (n = 6) or preparing to live apart (n = 2). Transitioning into separation was worse that the eventual separation itself. This is reflected in the gradual lessening in emotional intensity of the participants’ accounts as described their self-development in the ‘here and now’.

#### 3.1 The marital end: Crossing the Rubicon

Two participants decided to leave, but for the majority (n = 6) it was their husband who left. As a result, the participants had to ‘move on’; their marital thread. Their slender was now broken. Descriptive metaphors in the accounts, such as “a wake-up call” and “a turning point”, are indicative of a forced transition. Their marriage was over. This realisation seemed to be the end point of a process of separation, and varied across the accounts. Almost all felt a sense of the force of finality when they began to live apart: “The separation bit hadn’t fully kicked in when we were still living in the same house and he was still there for me. The ending really hit me hard then.” (Mary) Rose realised that her marriage was ‘really’ over after her husband began living with another man. Rose had dedicated herself to the vocation of marriage but her husband had broken the marital rules both by having an affair and by leaving. Relinquishing her attempt to regain control and accepting that her marriage was over was a shattering experience, but one which also brought relief and seemed to restore her clarity of thought. Her husband was gone:

When he left it was very, very difficult. Seeing all the missing things. He was already living with someone which was extremely painful for me. One evening when I called over and he didn’t open the door I became furious. I kicked the door and it shattered. I thought, ‘it’s over. I now pick up the pieces of my life and go on with my life’.

The thought of being and living alone, as an individual ‘self’, for the first time was coupled with trepidation and fear for the participants. This was foreign territory and a solo expedition. Having being married for so many years, several changes resulted from the mid-and later life separation, including threats to their emotional well-being, personal identity, and security (i.e. financial and residential). Sarah believed all her worries at this time related to her core concern, being alone and single: “I was worrying about the future. Will I have enough money, will we be ok? But looking back the worry really was just about being on my own. Missing the security.” A focus by others on the gay sexual orientation of her husband was additionally frustrating for her, and was experienced by her as minimising the significance of her loss. Sarah did not feel “lucky”; her marriage was not a success. Her husband had left her for someone else: “I’ve had loads of women saying to me ‘aren’t you lucky he left you for a man’. They don’t realise that it’s about the loss of the couple, and what that means for the family. What’s the difference what sex the person is. They still left you”.

#### 3.2 Self-integration: ‘Salvage what’s good and move on separately’

Cut off from the spousal relationship, a core source of support or focus, the participants moved towards taking control of their lives and created a new, meaningful identity. This was experienced as difficult, often painful, but rewarding. While most positive growth was gleaned from self-reflection and self-action, supportive friends and family members played a large part in sustaining the participants during their more difficult times. Feeling understood resulted in a sense of belonging and appeared to help redirect their focus on themselves. Many participants (n = 7) availed of therapeutic support when the cohabitation ended to help them to ‘return to themselves’. In contrast with previous therapeutic experiences, this was identified as helpful and often other, unexplored issues, such as family relationship issues, abuses and anxiety were also explored. Patty sought objective, if not directive, support to help her get to know, understand and be herself. She sought to break the pattern of focus on her husband, who had health issues. Entering her later life as single, she had to face the reality of returning to the workforce to secure her future. Her use of the word ‘I’ instead of ‘We’ reinforces her strengthening sense of self and singeldom.

When he said he was moving out I thought, ‘I need to take control of my life. I need a therapist; somebody objective to help me to do that. My default was, ‘how is my husband. Is he ok?’ That stopped me from feeling my own feelings, and thinking about practical, financial things.

The physical separation also resulted in unexpected perks (“now the toilet seat is always down!”) and unanticipated sexual experiences. All the participants highlighted the importance of transparency and trust in new relationships. Individual patterns emerged when they separated with some re-partnering and some remaining single. Words such as “foreign”, “cautious” and “daunting” were used to describe the initial concept of post-marital sex and process of exploration (n = 4). They did not want to be “hurt again”. Despite this concern, three participants reported happiness in living with another man, and one remarried. While Grace reported no desire to be with another man, she, like all the participants, strove to embrace unexplored parts of her life. Having her own space enabled her to return to her pre-marriage ‘self’, namely a dancer. Although the symbolic replacing of her husband ‘in the closet’ was indicative of a sense of tension and withdrawal, living apart was the liberating antithesis. Grace proudly took ownership of ‘creating a new life’, without judgement: “I had danced when I was young and I went back dancing. Now I have a whole network of people that know me. For a long time I felt that he came out and I went into the closet. I needed to get out. It has taken me years to get back to myself. I am not on edge anymore”.

Whilst Grace focused exclusively on herself in the present and future, most of the other participants accepted that their past experiences contributed to who they are now, and to their greatest gift, their children, and for some their grandchildren. Integrating their past and present self seemed to move them further towards psychological growth. Sarah described her attempts at integrating her past and present self, as she focuses on what she has achieved.

He could never be to me what I need, and I will never be to him what he needs. Salvage what is good, and move on separately. You have to move forward. I look back at the good—we have our children. Stay friends if you can. Just kept it simple and made it ok for the kids. You have to go the extra mile because of the prejudice they might get. Things happen to everyone. If you move beyond that and go forward with life then good things are there.

There is a sense of awareness that both her and her husband’s needs were better met outside their marriage. At the heart of Sarah’s identity as divorced are her past links to her marriage and shared parenting. Like most of the participants (n = 8), she continued to draw on insights from her past and expressed a desire to therapeutically support others in a similar situation. This seemed to further enhance a positive sense of self.

In summary, the participants, having for decades identified as married, had learned to make sense of a life separate from their husbands. This required a consequential shift in focus onto themselves. The realisation that there was no restoring of the past spurred participants into an appraisal process where their own abilities and others’ availability to them were assessed. The passage of time helped them to reflectively balance the loss of their marriage with the positive outcomes, namely their children and unexpected independent achievements. They rebuilt an integrated sense of self that was separate from, yet always somewhat connected to, their ex-husband by virtue of their children, and their history together.

## Discussion

This study focused on the experience of nine heterosexual women whose husbands came out as gay in mid-and later life. The loss of their marriage was extremely painful. Demonstrating the importance of the martial script to them, and concerns about social stigma, most tried to accommodate an altered marriage for a protracted period of time following the disclosure (being partially married, partially separated). In most cases it was the husband who finally left. Concerns regarding potential stigma towards them and their family were enhanced by virtue of their husband’s gay identity. Those who had positive communication with their husband experienced significant empathy towards him following the disclosure, which facilitated the resolution of the hurt incurred. Professional support sought following the disclosure was perceived to be judgmental, whilst therapy during or following their separation was experienced as supportive.

Marriage for the participants spoke to the internalisation of a traditional, monogamous script for coupledom, motherhood, and lifelong commitment. It was a permanent commitment in the cultural context of Catholicism. Marriage legitimised their relationship, and provided them with a plan for the future. Detaching from their ‘successful’ marriage following a perceived ‘lifetime’ (at least 15 years) of being interlinked with their husband was identified as ‘the most difficult thing’. Almost every participant who participated became tearful or cried when recalling the loss of their marriage. Most recalled fearing (hence presuming) societal stigma and being alone: “I did not want to be a divorcee”. In line with previous research, the women experienced the personal reactions commonly associated with a marital separation, such as stress, anger, fear and pain in processing the marital loss [35, 36, 37], concerns regarding their children, financial concerns and changes to their living arrangements [38, 39]. Being ‘older’ was an added consideration. The vulnerability and chance for poverty are higher for women post-divorce [40]. As with individuals separating or divorcing in mid- and later life, many felt more economically vulnerable and reported a lack of confidence and uncertainty regarding their futures [41]. Breaking the dependency on one another and negotiating the reality of their situation was complex. Illness for some of the participants or that of their husband made separating additionally difficult given the increased need for care. This is in keeping with research highlighting the positive link between increasing age and the risk of illness and associated care needs [12, 42, 43].

An enhanced awareness of societal sexual prejudice relating to the disclosure of a gay sexual orientation that is highlighted in previous studies (e.g. [25, 26]) was also experienced by these women. They had grown up in a society where homosexual marriage was illegal; legislation allowing for same sex marriage is a twenty first century phenomenon. For many, in seeking to protect the self and family unit, there were dilemmas about when and to whom to disclose the reason for their marriage difficulties or separation, namely the ‘coming out’. The women wanted to share their experiences with others who they felt would understand. They found it difficult to deal with dismissive suggestions to quickly separate or ‘move on’ if the couple did not wish to separate, or the assumption that the experience of marital loss should be experienced less intensely because of the gay sexual orientation of their spouse: “at least he is not leaving you for a woman”. While being ‘out’ (the degree to which others know the sexual orientation of the self and others) is linked to increased relationship quality [44], the findings show that such benefits did not apply to their marriage.

Most of the women had tried to sustain and accommodate a state of being partially married and partially separated, but it created tension for them. Perceived bias from professional therapists during couple therapy at the time of the disclosure was experienced as additionally isolating, especially for those for whom informal support (family, friends, on-line fora) was limited. The findings concur with research on the negative psychological consequences that can occur if individuals perceive themselves as being ‘alone’ in processing a significant loss or societal stigma [45]. Conversely, non-judgmental support from others that focused on the women’s needs and wants was perceived as helpful. In order to cope with the demanding process of finally ‘uncoupling’, most of the women then sought support from a professional therapist. In contrast with their previous therapeutic experiences, this latter support was experienced as beneficial and helped them to perceive the life transition as an opportunity for personal development. Similar positive health benefits are reflected in the literature on social and therapeutic support, with reductions cited in distorted thinking and conflict [46, 47]. It may be that therapeutic practices have improved in recent years and that the women had more efficacy and control of their lives at this later stage–they could, and had to, focus on themselves. It may also be that this experience is being increasingly integrated into mainstream conversations about sexuality and marriage, in addition to the existence of several on-line support groups. Nevertheless, our findings emphasise the importance of therapeutic neutrality, and of educators and therapists being aware of their own attitudes and beliefs about relationships, infidelity, sexual orientation and divorce [48].

All the women had to cope with their “shattered assumptions” [49], re-conceptualise the self, and reconstruct their life narrative [50]. This involved constructing a ‘new’ identity around the concept of being separated or divorced (e.g. having an ‘ex’, post-separation co-parenting, being single or repartnering). By engaging in self-action, such as solo pursuits, independent living and new relationships, they began to transition to a self-image as fully separated. The transition involved uncertainty and an acknowledgment, as seen in literature on therapeutic change, that they were moving away from a position of certainty of how things should be (the marital script), and “towards positions that entertain different possibilities” ([51]p195). Constructing a new self-identity was breaking the gender norms they had attempted to uphold in their previous marital relationship (e.g. pressure to marry for life and being homemakers/caregivers). This highlights the fluid, dynamic and contextual nature of identity [52]. There was no finality to their past lives, by virtue of their shared connection with their children. Moreover, many of the women continued to positively connect with their ex-husbands, integrating their past and present self. For all, the disruption that co-occurs with a marital separation, stabilised and moderated over time [35, 53]. Therapeutic tools, such as autobiography and narrative therapy, may further help deconstruct a distressing story and ‘reauthor’ a meaningful, alternative one [54].

One of the most significant findings, which contributes to the extant literature, was the presence and role of empathy towards their husbands in enabling the women to reconnect with them and to eventually forgive them for the injury incurred by the broken marriage. This related exclusively to the women who reported positive communication with their husband prior to, and following, the disclosure, and whose husband had shared their remorse regarding the suppression and disclosure of their gay sexual orientation with them. While accepting responsibility was synonymous with bearing blame, it was not ‘fully’ their husband’s fault, given the homophobic culture in which they had grown up [55, 56]. Initially the empathetic connection and concern for their husband made it additionally difficult for most of the women to ‘move on’. They felt with, and possibly even for, their husband rather than feeling against them. It also seemed to enhance the communication between the couple, thereby facilitating resolution of the hurt at the marital dissolution, and constructive change.

This finding is in keeping with research on the process of forgiveness which highlights the importance of empathy in being able to emotionally recover following an interpersonal injury [57, 58]. It involves a process of transforming feelings, as well as giving up the hope that the past (or person) can be changed [59] following a protracted period of time trying to do so. This was borne out by the participants who transformed feelings from hurt and anger to compassion, and (over time) had to focus on their own future. It should be noted that most of the participants recalled having a happy marriage, prior to the disclosure, and a positive relationship with their husband. This is likely to have been a factor in their ability to restore amicable closeness with their husband given that forgiveness has been shown to occur more frequently in the context of close relationships [60].

### Limitations

There are a number of limitations in the current study. Firstly, regarding recruitment, a selection bias may have occurred because of the voluntary nature of the recruitment. Those who participated may have been more comfortable in discussing their private lives with the researcher or in accessing support. Secondly, the accounts were retrospective in nature and they, as with all retrospective research, need to be interpreted with caution. The accounts may have been influenced by memory bias and the affective state of each participant at the time of interview. Finally, the results obtained from the data are not generalisable due the limitations of small size, the characterisation of the sample population, and the nature of IPA. The accounts of heterosexual husbands were excluded, given the necessary homogeneity of our sample. However, the objective of qualitative research is concerned with the quality of experiences, rather than the identifying cause-effect relationships. The results of this study are specific to the perceptions and context of the particular participants who partook, and are, therefore, more suggestive rather than conclusive.

### Conclusions

Our findings contribute to the literature on the experience of a husband coming out as gay, and are novel in terms of the systematic research method employed, the focus on the process of marital separation, and the impact of culture and empathy on the resolution process. While there was some divergence across the accounts, the majority of the women emphasised marital separation and the process of negotiating loss of the marriage as more traumatic than the husband’s gay identity. Nonetheless, an appreciation of the individuality and cultural context of each marriage and person was, and is, required. Separating involved a diverse process of coming to see the self as a separated and single person, and mourning the loss of a marital identity into which they had invested so deeply. The women in this study demonstrated that a husband coming out as gay can mean a long marital goodbye, an immediate separation, or a continued marriage. All involve varying degrees of pain and loss, and a focus on the separate self and self-care can provide a pathway to healing.
